# Association Between the Frailty Index and Self‐Reported Headache in Middle‐Aged and Older Chinese Adults: An Analysis of the China Health and Retirement Longitudinal Study

**DOI:** 10.1155/prm/6632755

**Published:** 2026-07-14

**Authors:** Mixue Guo, Qixin Chen, Huqiang Dong, Zufa Zhang, Yuanbin Huang, Xiaoming Li

**Affiliations:** ^1^ School of Basic Medical Sciences, Ningxia Medical University, Yinchuan, 750004, Ningxia, China, nxmu.edu.cn; ^2^ Department of Oral Implantology, The Affiliated Stomatological Hospital of Xuzhou Medical University, Xuzhou, 221003, Jiangsu, China; ^3^ School of Public Health, Ningxia Medical University, Yinchuan, 750004, Ningxia, China, nxmu.edu.cn; ^4^ Department of Urology, Affiliated Zhongshan Hospital of Dalian University, Dalian, 116001, Liaoning, China, dlut.edu.cn; ^5^ Division of Pancreatic Surgery, Department of General Surgery and Laboratory of Pancreatic Cancer, West China Hospital, Sichuan University, Chengdu, 610041, Sichuan, China, scu.edu.cn; ^6^ West China School of Medicine, Sichuan University, Chengdu, 610041, Sichuan, China, scu.edu.cn

**Keywords:** CHARLS, cross-sectional study, frailty index, headache, older adults

## Abstract

**Background:**

Headache is one of the common subjective discomfort symptoms in middle‐aged and elderly people, which seriously affects the quality of life. Frailty is a syndrome characterizing the aging state, but the association between frailty and headache lacks support from large‐sample studies.

**Methods:**

This cross‐sectional analysis used data from the China Health and Retirement Longitudinal Study (CHARLS). We concatenated the 2011/2013/2015 waves and used cluster‐robust standard errors at the participant level to account for repeated measures; all analyses applied CHARLS sampling weights. A 35‐item index was used to construct the frailty index (FI) and grouped by quartiles. The association between frailty and headache was assessed using multifactorial logistic regression models, and nonlinear associations were assessed using restricted cubic spline (RCS) analysis. Robustness tests included subgroup analyses and interaction tests.

**Results:**

A total of 28,402 participants were included. Multivariable logistic regression showed that FI was positively associated with headache. In the fully adjusted model, each 0.1‐point increase in FI was associated with a 13.5% higher odds of headache (OR = 1.135, 95% CI: 1.130–1.139, *p* < 0.001). Compared with Q1, participants in Q2, Q3, and Q4 had progressively higher odds of headache, with the highest odds observed in Q4 (P for trend < 0.001). RCS analysis indicated a significant nonlinear positive association between FI and headache (P for nonlinearity < 0.001), with a more pronounced increase in headache odds at higher FI levels. Subgroup analyses were generally consistent, with nominal interactions observed by drinking status and hypertension.

**Conclusion:**

Higher frailty levels were associated with greater headache odds with a nonlinear pattern. Given the cross‐sectional design, temporality and causality cannot be established, and future longitudinal studies are needed to clarify causal relationships.

## 1. Introduction

With the increasing trend of population aging, frailty has become an important public health problem affecting the physical and mental health and quality of life of middle‐aged and elderly people [1, 2]. Frailty is usually defined as a clinical syndrome of multidimensional, progressive decline characterized by decreased physiological reserve function and elevated vulnerability to external stressors [3, 4]. It has been shown to significantly increase the risk of adverse outcomes such as falls, hospitalization, and death [5–7]. The frailty index, constructed based on the cumulative model of health deficits, integrates information from multiple dimensions of an individual’s physical, psychological, and social functioning to provide a continuous score, and is currently a widely used, highly sensitive, and stable tool to quantify frailty [8–10]. Studies have further found that frailty is not only significantly associated with dysfunction and mortality, but also has a strong association with a variety of chronic symptoms, such as chronic pain, depression, sleep disorders, and cognitive impairment [11–14].

Headache, as a common subjective symptom, has garnered significant attention among middle‐aged and elderly populations in recent years. Epidemiological studies indicate that a substantial proportion of this demographic experiences persistent or recurrent headaches, particularly chronic tension‐type headaches [15, 16]. This type of headache is often intertwined with factors such as shoulder and neck muscle tension, psychological stress, and sleep disturbances, significantly impacting quality of life [17, 18]. According to the Global Burden of Disease Study, migraine and tension‐type headache consistently rank among the leading causes of disability worldwide [19, 20]. Headaches are generally classified as primary (e.g., migraine, tension‐type headache) or secondary (e.g., intracranial lesions, infections), with primary types being more prevalent [21–23]. Although the overall prevalence of headaches among middle‐aged and elderly individuals is lower than that among young and middle‐aged adults, it exhibits characteristics such as chronicity, strong insidiousness, and high intractability [24, 25]. Tension‐type headaches, for example, often present as persistent dull pain. They frequently overlap with factors such as neck and shoulder muscle tension, psychological stress, and sleep disturbances, leading to neglect or misdiagnosis [26–28]. More importantly, headaches as a complex health issue often coexist with psychosocial factors such as depression, anxiety, cognitive impairment, and chronic fatigue, particularly among older adults experiencing multiple comorbidities and physical functional decline [29–31]. This population exhibits heightened sensitivity to pain perception, making them prone to developing chronic headache patterns, thereby increasing the overall health burden [18, 32].

There are still few studies on whether frailty increases the risk of headache, and some studies have found that headache may be closely related to factors such as anxiety, depression, and poor sleep quality, which are also more common in frail populations [33]. Based on this, the present study was designed to construct a frailty index based on 35 health deficits using nationally representative data from the China Health and Retirement Longitudinal Study (CHARLS) to systematically assess the level of frailty in the middle‐aged and elderly population and to investigate the association between frailty and headache. This study is expected to provide new ideas and empirical evidence for the screening and intervention of headache in the middle‐aged and elderly population.

## 2. Materials and Methods

### 2.1. Study Population

This study is based on the CHARLS database. CHARLS is a nationwide, longitudinal survey of people aged 45 years and older in mainland China that aims to systematically assess the social, economic, and health status of middle‐aged and older adults. Launched in 2011, the survey employs a stratified, multistage, probability proportional to size sampling strategy, and covers urban and rural communities in 28 provinces across China, making it highly representative. CHARLS uses a standardized questionnaire and uniformly trained enumerators to collect multidimensional information, including demographic characteristics, history of chronic diseases, and other information through face‐to‐face interviews. The database has been widely used for research in the fields of healthy aging and chronic disease management, and is highly recognized internationally.

This study is the first to explore the association between frailty and headache using CHARLS data. All participants signed a written informed consent form before the investigation, and the research project was approved by the Medical Ethics Review Board of Peking University (IRB00001052‐11015) and was conducted in strict compliance with the Declaration of Helsinki and related ethical norms. The study design and data acquisition instructions have been reported in detail in the preliminary literature, and publicly available data can be accessed through the official website (https://charls.pku.edu.cn) [34, 35].

**FIGURE 1:**
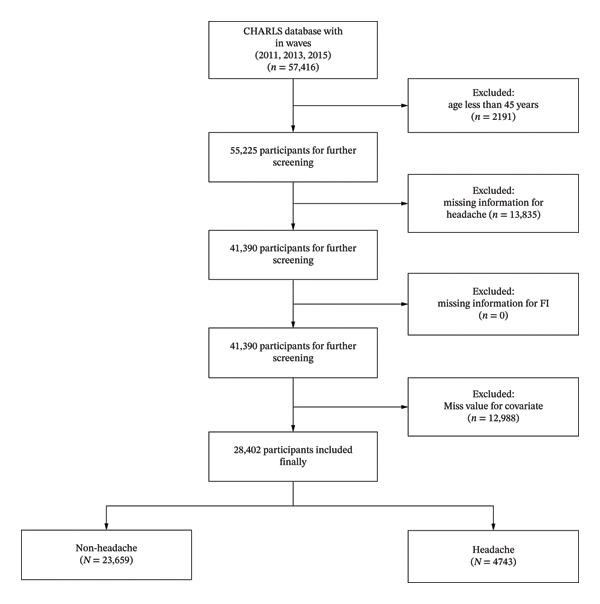
Participant selection flowchart.

According to the exclusion process outlined in Figure 1, the preliminary screening identified a total of 57,416 headache patients in the CHARLS database, with data sourced from the 2011, 2013, and 2015 survey waves. During screening, 2191 participants under age 45, 13,835 participants with missing headache data, and 12,988 participants with missing covariate data were excluded. Participants with missing data in any of the 35 FI components were excluded. In contrast, participants with a calculated FI value of 0 were retained, because FI = 0 indicated that none of the included health deficits was present and did not represent missing FI data. Ultimately, 28,402 participants meeting inclusion criteria were enrolled: 23,659 in the Non‐Headache group and 4743 in the Headache group (Figure 1). We further compared the baseline characteristics of included and excluded participants to assess potential selection bias due to missing data, and the results are shown in Table S1.

### 2.2. Assessment of Headache

The CHARLS survey collects headache information from participants through self‐assessment. In the “Physical Health Status” module, participants were asked to answer the question “Do you suffer from physical pain? (Do you feel pain often?)”. If the answer was “yes,” the question was followed up with “Where do you experience pain?” Based on the responses, this study identified participants who reported “headache” as the site of their headache and defined them as suffering from headache. Since the CHARLS does not further categorize headache types (such as migraine or tension‐type headache), this study collectively referred to them as “headache” as a dichotomous variable (yes/no) [36].

### 2.3. Assessment of Frailty Index

This study employs the cumulative deficit model to construct a frailty index for quantifying frailty levels in middle‐aged and elderly populations. The FI is calculated by summing an individual’s scores across all health deficits, then dividing this sum by the total number of deficits included. This yields a continuous variable ranging from 0 to 1, where higher FI values indicate greater frailty. Health deficits here refer to any indicators of physical or psychological impairment that emerge with age and may increase the risk of adverse health outcomes. The FI incorporates 35 health deficits across multiple dimensions, including items related to activities of daily living/instrumental activities of daily living, physical functional limitations, chronic diseases, mental health, and subjective health assessments. This provides a comprehensive reflection of an individual’s multidimensional health status. For variable coding, each deficit was assigned a score ranging from 0 to 1, where 0 indicated the absence of a deficit and 1 indicated the presence of the most severe deficit. For variables with ordered response categories, intermediate values such as 0.25, 0.33, 0.5, 0.67, or 0.75 were assigned according to the severity of the response category, as detailed in Supporting Table S2 [37]. To ensure comparability, FI was computed only for participants with complete information on all 35 deficits; individuals with missing data in any FI component were excluded from FI computation and the analytic sample. Importantly, we verified that none of the 35 deficits referred to “body pain,” “head pain,” “headache,” or any synonyms; therefore, FI does not incorporate any component of the outcome, and there is no circularity by construction.

### 2.4. Covariates

The covariates in this study included age, sex, BMI, marital status, education level, residence, smoking status, drinking status, history of hypertension, history of diabetes mellitus, and dyslipidemia. Sex was categorized as male and female; marital status as married and nonmarried (including divorced, widowed, and never married); education level as illiterate, elementary/junior high school, and high school and above; residence as urban and rural; smoking status as whether or not the individual smoked; drinking status as whether or not the individual consumed alcohol; history of hypertension and diabetes mellitus as whether or not the individual had ever been diagnosed with these conditions.

### 2.5. Statistical Analyses

In this study, continuous variables were expressed as mean ± standard deviation and categorical variables were expressed as frequency (percentage) based on the normality of data distribution. The population was categorized into four groups based on frailty quartiles. Quartile‐based categorization was used to ensure balanced group sizes and to examine the dose–response pattern across the FI distribution; this approach is intended for risk‐gradient comparison rather than clinical frailty classification based on fixed cut‐offs. Differences between continuous variables were assessed using analysis of variance or Kruskal–Wallis test, and differences between categorical variables were compared using chi‐square test. To assess the association between frailty and headache occurrence, analyses were performed using logistic regression (OR) and its 95% confidence interval (CI), with stepwise inclusion of covariates to control for potential confounders. Model 1 was not adjusted for any covariates, and model 2 was adjusted for age, sex, residence, marital status, education level, and BMI on the basis of model 1. Model 3 was further adjusted for smoking status, drinking status, hypertension, diabetes mellitus, and dyslipidemia on the basis of model 2. And the association between FI and headache was assessed using the trend test (P for trend). To evaluate whether the large effect estimate for the highest FI quartile was driven by participants with extremely high frailty levels, we performed a sensitivity analysis after excluding participants with FI values above the 95th percentile. The same logistic regression models were then refitted in the remaining sample, and FI quartiles were recalculated in the sensitivity analysis sample. To further explore the association between frailty and headache, models were fitted using the RCS function. Subgroup analysis and interaction tests were conducted to assess the heterogeneity of the association between FI and headache across different demographic and clinical subgroups. A total of 15 comparisons were performed, with an adjusted significance threshold of *α*′ = 0.05/15 ≈ 0.0033. For the interaction tests, statistical significance after Bonferroni correction was defined as a *p* value below this adjusted threshold. Note that Bonferroni correction is a conservative method that may increase the risk of Type II errors. Therefore, the findings should be interpreted with caution, and further studies are needed to validate these subgroup effects. All statistical analyses were performed using R statistical software (version 4.4.0). A two‐sided *p* value < 0.05 was considered statistically significant. This study was reported according to the STROBE guidelines, and the completed checklist is provided in Table S4

## 3. Results

### 3.1. Baseline Characteristics of the Study Population

Based on baseline characteristics in Table 1, the study sample comprised 28,402 participants divided into four groups according to FI quartiles. Participants’ age significantly increased with rising frailty index scores. The proportion of women significantly increased in the high‐frailty group (*p* < 0.001). BMI showed minor but statistically significant differences across groups. The proportion of unmarried individuals increased with greater frailty, while higher educational attainment was predominantly concentrated in the low‐frailty group. The proportion of rural residents increased with greater frailty, rising from 55.15% in Q1 to 69.52% in Q4 (*p* < 0.001). Smoking and alcohol consumption rates were lower in the high‐frailty group. Prevalence of hypertension and diabetes mellitus significantly increased with greater frailty (*p* < 0.001). Additionally, dyslipidemia was more prevalent in the high‐frailty group (*p* < 0.001). Most notably, headache incidence significantly increased with rising frailty scores (*p* < 0.001) (Table 1).

**TABLE 1:** Baseline characteristics of the study population based on FI quartiles.

Characteristics	Total (*n* = 28,402)	Q1 (*n* = 7819)	Q2 (*n* = 6344)	Q3 (*n* = 7089)	Q4 (*n* = 7150)	*p* value
Age (years)	60.02 ± 9.52	57.39 ± 8.56	58.81 ± 9.08	60.36 ± 9.46	63.63 ± 9.79	< 0.001
Sex (%)						< 0.001
Female	15,465 (54.45)	3407 (43.57)	3250 (51.23)	4109 (57.96)	4699 (65.72)	
Male	12,937 (45.55)	4412 (56.43)	3094 (48.77)	2980 (42.04)	2451 (34.28)	
BMI	23.75 ± 3.80	23.97 ± 3.50	23.80 ± 3.70	23.64 ± 3.86	23.59 ± 4.12	< 0.001
Marital status (%)						< 0.001
Married	24,760 (87.18)	7189 (91.94)	5650 (89.06)	6177 (87.13)	5744 (80.34)	
Nonmarried	3642 (12.82)	630 (8.06)	694 (10.94)	912 (12.87)	1406 (19.66)	
Education (%)						< 0.001
High school or above	3343 (11.77)	1437 (18.38)	833 (13.13)	689 (9.72)	384 (5.37)	
Illiterate	7529 (26.51)	1313 (16.79)	1446 (22.79)	1963 (27.69)	2807 (39.26)	
Junior high school	17,530 (61.72)	5069 (64.83)	4065 (64.08)	4437 (62.59)	3959 (55.37)	
Residence (%)						< 0.001
Rural	17,576 (61.88)	4312 (55.15)	3751 (59.13)	4542 (64.07)	4971 (69.52)	
Urban	10,826 (38.12)	3507 (44.85)	2593 (40.87)	2547 (35.93)	2179 (30.48)	
Smoke (%)						< 0.001
No	20,781 (73.17)	5268 (67.37)	4511 (71.11)	5293 (74.66)	5709 (79.85)	
Yes	7621 (26.83)	2551 (32.63)	1833 (28.89)	1796 (25.34)	1441 (20.15)	
Drink (%)						< 0.001
No	18,962 (66.76)	4479 (57.28)	4039 (63.67)	4926 (69.49)	5518 (77.17)	
Yes	9440 (33.24)	3340 (42.72)	2305 (36.33)	2163 (30.51)	1632 (22.83)	
Hypertension (%)						< 0.001
No	16,231 (57.15)	4937 (63.14)	3884 (61.22)	4017 (56.67)	3393 (47.45)	
Yes	12,171 (42.85)	2882 (36.86)	2460 (38.78)	3072 (43.33)	3757 (52.55)	
Diabetes mellitus (%)						< 0.001
No	24,613 (86.66)	7066 (90.37)	5597 (88.23)	6155 (86.82)	5795 (81.05)	
Yes	3789 (13.34)	753 (9.63)	747 (11.77)	934 (13.18)	1355 (18.95)	
Dyslipidemia (%)						< 0.001
No	20,382 (71.76)	5906 (75.53)	4686 (73.87)	5044 (71.15)	4746 (66.38)	
Yes	8020 (28.24)	1913 (24.47)	1658 (26.13)	2045 (28.85)	2404 (33.62)	
Headache (%)						< 0.001
No	23,659 (83.30)	7654 (97.89)	5957 (93.90)	5969 (84.20)	4079 (57.05)	
Yes	4743 (16.70)	165 (2.11)	387 (6.10)	1120 (15.80)	3071 (42.95)	

*Note:* Mean ± SD for continuous variables: the *p* value was analyzed via ANOVA. (%) for categorical variables: the *p* value was analyzed via the chi‐square test.

### 3.2. Association Between FI and Headache

In Table 2, FI was first analyzed as a continuous variable. In the fully adjusted model, each 0.1‐point increase in FI was associated with a 13.5% higher odds of headache (OR = 1.135, 95% CI: 1.130–1.139, *p* < 0.001). In the quartile‐based analysis, the odds of headache increased progressively across FI quartiles. In Model 3, compared with Q1, the ORs for Q2, Q3, and Q4 were 2.92, 8.06, and 35.09, respectively, and the trend was statistically significant (P for trend < 0.001). The positive association between FI and headache was consistent across all models (Table 2). To assess the influence of extreme frailty values, we further excluded participants with FI values above the 95th percentile. The association remained robust in the fully adjusted model, with each 0.1‐point increase in FI associated with higher odds of headache (OR = 1.185, 95% CI: 1.178–1.192, *p* < 0.001). Compared with Q1, the OR for Q4 was attenuated but remained significant (OR = 27.056, 95% CI: 22.915–31.946, *p* < 0.001), with a significant trend across FI quartiles (P for trend < 0.001) (Table S3). Because this sensitivity analysis was based on a truncated sample after removal of the highest 5% of FI values and did not recalibrate survey weights for the altered sample structure, these estimates should be interpreted as a robustness check and may not be directly generalizable to the most frail individuals.

**TABLE 2:** Multiple logistic regression analysis of the association between FI and headache.

Characteristic	Model 1		Model 2		Model 3	
	OR (95% CI)	*p*‐value	OR (95% CI)	*p*‐value	OR (95% CI)	*p*‐value
FI	1.129 (1.124, 1.133)	< 0.001	1.135 (1.130, 1.139)	< 0.001	1.135 (1.130, 1.139)	< 0.001
FI groups						
Q1	Reference		Reference		Reference	
Q2	3.04 (2.54, 3.63)	< 0.001	2.94 (2.45, 3.52)	< 0.001	2.92 (2.44, 3.50)	< 0.001
Q3	8.47 (7.20, 9.97)	< 0.001	8.15 (6.92, 9.61)	< 0.001	8.06 (6.84, 9.50)	< 0.001
Q4	35.01 (29.94, 40.93)	< 0.001	35.83 (30.52, 42.07)	< 0.001	35.09 (29.88, 41.22)	< 0.001
*p* for trend		< 0.001		< 0.001		< 0.001

*Note:* For continuous FI, ORs are presented per 0.1‐point increase. Model 1: No covariates were adjusted. Model 2: Age, sex, residence, marital status, educational attainment, and BMI were adjusted. Model 3: Age, sex, residence, marital status, educational attainment, BMI, smoking status, drinking status, hypertension, diabetes mellitus, and dyslipidemia were adjusted.

Abbreviation: FI, frailty index.

### 3.3. Restricted Cubic Spline Analysis of FI and Headache

**FIGURE 2:**
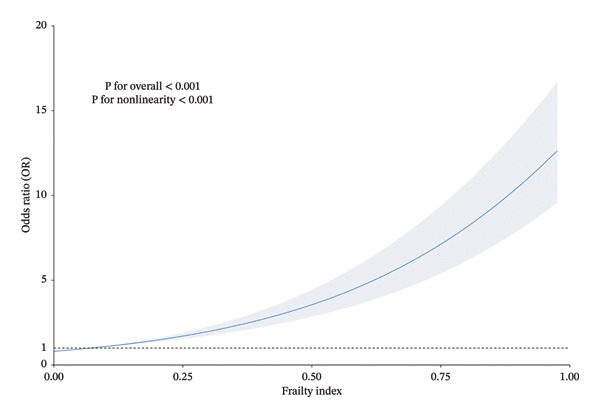
Restricted cubic spline analysis of the association between the frailty index and headache in the fully adjusted model. Note: The odds ratio was calculated using FI = 0.11 as the reference value, corresponding to OR = 1. The solid line represents the adjusted odds ratio, and the shaded area represents the 95% confidence interval. The horizontal dashed line indicates OR = 1. The model was adjusted for age, sex, residence, marital status, educational attainment, BMI, smoking status, drinking status, hypertension, diabetes mellitus, and dyslipidemia.

Restricted cubic spline analysis was used to examine the potential nonlinear association between FI and headache. As shown in Figure 2, the adjusted odds of headache increased progressively with higher FI levels, and the increase appeared to be more pronounced at higher levels of frailty. The overall association was statistically significant, and the test for nonlinearity was also significant (P for nonlinearity < 0.001), supporting a nonlinear positive association between FI and headache.

### 3.4. Subgroup Analyses and Interaction Tests

**FIGURE 3:**
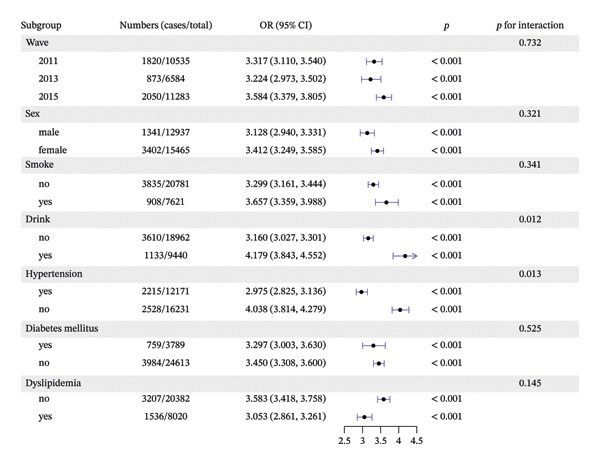
Investigation of the association between frailty and headache by subgroup. Note 1: age, sex, residence, marital status, educational attainment, BMI, smoking status, drinking status, hypertension, diabetes mellitus, and dyslipidemia were all taken into account while adjusting the aforementioned model. Note 2: The model does not account for the stratification variable in any of these situations. Note 3: A total of 15 interaction tests were performed. After Bonferroni correction, the adjusted significance threshold was *α*′ = 0.05/15 ≈ 0.0033. The interactions for drinking status and hypertension were nominally significant but did not remain statistically significant after Bonferroni correction.

Because 15 interaction tests were performed, the Bonferroni‐corrected significance threshold was set at *α*′ = 0.05/15 ≈ 0.0033. To further explore the heterogeneity in the association between FI and headache, subgroup analyses and interaction tests were conducted across demographic, lifestyle, and clinical subgroups. The positive association between FI and headache was generally consistent across most subgroups. Nominal interactions were observed for drinking status and hypertension, with *p* values for interaction of 0.012 and 0.013, respectively. However, these interaction *p* values did not meet the Bonferroni‐corrected threshold of 0.0033 and thus should be considered exploratory rather than confirmatory. Therefore, the subgroup findings should be interpreted as exploratory and hypothesis‐generating (Figure 3).

## 4. Discussion

This study, based on nationally representative CHARLS data among middle‐aged and older adults, systematically examined the association between FI and headache. We found that higher FI was significantly and positively associated with headache, and this association remained robust after adjusting for demographic factors, lifestyle behaviors, and chronic conditions. A clear dose–response pattern was observed whether FI was modeled as a continuous variable or categorized into quartiles (P for trend < 0.001). In the nonlinear analysis, we focused on the RCS results rather than estimating a specific clinical threshold, because the aim was to evaluate the overall shape of the association between FI and headache. The RCS curve suggested that headache odds increased progressively with FI, particularly at higher frailty levels. The large effect estimate observed in the highest FI quartile should be interpreted cautiously. In the sensitivity analysis excluding participants with FI values above the 95th percentile, the association remained significant, although the OR for Q4 versus Q1 was attenuated. This suggests that the main findings were not solely driven by extreme FI values. Nevertheless, residual confounding, symptom clustering, measurement limitations, and reverse causality may still partly explain the large association.

The mechanisms underlying the association between frailty and headache are likely multifactorial and should be interpreted in the context of the FI used in this study. The FI does not capture a single pathological process but rather reflects the accumulation of multidimensional health deficits across physical function, chronic diseases, mental health, and self‐rated health [8–10, 37]. Therefore, the observed association is unlikely to be explained by one isolated biological pathway. Instead, it may reflect the combined effects of reduced physiological reserve, systemic inflammation, vascular dysfunction, altered pain processing, and psychosocial vulnerability.

Alcohol use may provide an important example of how reduced physiological reserve could modify the frailty–headache association. Previous studies have shown that alcohol consumption is associated with frailty risk and may influence pain sensitivity and headache occurrence [38, 39]. Alcohol can affect headache through neurovascular changes, sleep disruption, dehydration, and altered pain sensitivity [40]. In frail individuals, diminished homeostatic capacity and possible age‐related changes in pharmacokinetics and pharmacodynamics may increase vulnerability to these effects [41, 42]. Therefore, the observed interaction with drinking status may not necessarily indicate a direct causal role of alcohol, but rather suggests that alcohol‐related physiological stressors may have a stronger impact among individuals with higher frailty burden.

Vascular dysfunction may be particularly relevant to the present findings, especially in light of the observed interaction with hypertension. Hypertension is commonly associated with endothelial dysfunction, arterial stiffness, impaired cerebral autoregulation, and altered cerebral perfusion [43]. In frail individuals, reduced physiological reserve and accumulated vascular damage may make the brain more vulnerable to fluctuations in blood pressure and cerebral blood flow. This may contribute to abnormal activation of pain‐sensitive neurovascular structures and increase susceptibility to headache. From this perspective, hypertension may represent a vascular phenotype through which frailty‐related multisystem dysregulation is linked to headache risk [44].

Inflammation and altered pain processing may also contribute to the observed association. Frailty has been closely linked to chronic low‐grade inflammation, sometimes referred to as inflammatory aging [45–47]. Pro‐inflammatory mediators such as IL‐1β, IL‐6, and TNF‐α may facilitate trigeminovascular activation, neurogenic inflammation, and sensitization of pain pathways, thereby contributing to headache susceptibility [48–50]. In parallel, neurovascular coupling dysfunction may also play a bridging role between frailty and headache [51, 52]. In frail populations, reduced arterial elasticity, vascular endothelial impairment, and chronic hypoperfusion may disturb the dynamic regulation of cerebral blood flow, resulting in a mismatch between cerebral metabolic demand and blood supply [53–56]. Moreover, peripheral inflammatory signals may promote microglial activation and central sensitization, thereby lowering the threshold for pain perception or increasing the persistence of headache symptoms [57–59]. However, because this study was cross‐sectional and did not include inflammatory biomarkers, vascular function measures, neuroimaging indicators, or detailed headache phenotyping, these mechanisms remain hypothetical and should be examined in future longitudinal and mechanistic studies.

## 5. Strengths and Limitations

To the best of our knowledge, this is the first study to examine the association between frailty and headache in a large sample. The strengths of this study are the inclusion of a nationally representative sample of the Chinese middle‐aged and elderly population, the adjustment for multiple covariates, and the robustness of the findings.

Although this study was based on a nationally representative sample and included a large number of middle‐aged and older adults, several limitations should be acknowledged. First, headache was assessed using self‐reported questionnaire data, and CHARLS did not distinguish between specific headache types, such as migraine, tension‐type headache, medication‐overuse headache, and secondary headache. Therefore, outcome misclassification is possible. Such misclassification is likely to be nondifferential with respect to FI and may therefore bias the association toward the null; however, the lack of headache phenotyping limits the pathophysiological interpretation of our findings. Second, frailty was assessed using an FI constructed from variables available in CHARLS. Although the FI is a widely used and sensitive measure of accumulated health deficits, some objective performance‐based indicators, such as gait speed, grip strength, and other physical performance tests, were not available or not included in the present FI construction. The omission of these measures may affect the completeness of frailty assessment and may limit the comparability of our results with studies using phenotype‐based frailty definitions. Third, although we adjusted for a range of demographic characteristics, lifestyle factors, and chronic conditions, residual confounding cannot be fully excluded. In particular, detailed psychiatric history, depression severity, anxiety symptoms, sleep quality, socioeconomic stress, medication use, and polypharmacy were not comprehensively accounted for in the present analysis. These unmeasured or incompletely measured factors may have contributed to the large effect estimates observed for the highest FI quartile. Fourth, due to differences in certain baseline characteristics between included and excluded participants, selection bias may have been introduced. Future studies using multiple imputation, inverse probability weighting, or other missing‐data methods may help evaluate the robustness of the findings. Fifth, the sensitivity analysis excluding FI values above the 95th percentile reduced the influence of extreme frailty values but was performed on a truncated sample without recalibrating the survey weights or fully accounting for the altered population structure. Therefore, the sensitivity analysis results should be interpreted as robustness evidence only and may not be directly generalizable to the most frail individuals. Finally, because of the cross‐sectional design, temporality and causality cannot be established. Reverse causality is a particularly plausible alternative explanation. Chronic or recurrent headache may lead to reduced physical activity, sleep disturbance, depressive symptoms, functional decline, and poorer self‐rated health, which could in turn increase FI. Therefore, longitudinal studies are needed to determine whether frailty increases the risk of headache, headache contributes to frailty progression, or both processes coexist bidirectionally.

## 6. Conclusion

This study found that frailty is positively associated with the risk of headaches in middle‐aged and older adults. Although this research reveals an important association, its cross‐sectional design precludes establishing a causal relationship between the two. Therefore, future studies should further investigate the causal relationship between frailty and headaches to provide a more scientific basis for headache prevention and intervention in middle‐aged and older adults.

**Nomenclature**
FI: Frailty index
CHARLS: China Health and Retirement Longitudinal Study
CI: Confidence interval
OR: Odds ratio
RCS: Restricted cubic spline

## Author Contributions

Mixue Guo and Qixin Chen designed the research. Zufa Zhang and Huqiang Dong collected and analyzed the data and drafted the manuscript. Yuanbin Huang and Xiaoming Li revised the manuscript.

## Funding

This work did not receive any specific grant from any funding agency in the public, commercial, or not‐for‐profit sector.

## Disclosure

All authors contributed to the article and approved the submitted version.

## Ethics Statement

The CHARLS study was performed in accordance with the principles of the Declaration of Helsinki and was approved by the Institutional Review Board of Peking University (IRB00001052–11015). All participants provided written informed consent before participating in the CHARLS study.

## Consent

The authors have nothing to report.

## Conflicts of Interest

The authors declare no conflicts of interest.

## Supporting Information

Additional supporting information can be found online in the Supporting Information section.

## Supporting information


**Supporting Information** Table S1. Baseline characteristics of participants included in and excluded from the missing‐data comparison sample. Table S2. List of health deficit items included in the frailty index. Table S3. Sensitivity analysis of the association between FI and headache after excluding participants with FI values above the 95th percentile. Table S4. STROBE Statement—checklist of items that should be included in reports of observational studies.

## Data Availability

The data that support the findings of this study are available from the China Health and Retirement Longitudinal Study (CHARLS) repository. Access to these data can be obtained by registering and submitting a request through the official CHARLS website at https://charls.pku.edu.cn.

## References

[1] Searle S. D. and Rockwood K. , What Proportion of Older Adults in Hospital are Frail?, The Lancet. (2018) 391, no. 10132, 1751–1752, 10.1016/s0140-6736(18)30907-3. 29706363

[2] Kojima G. , Taniguchi Y. , Iliffe S. , Urano T. , and Walters K. , Factors Associated With Improvement in Frailty Status Defined Using the Frailty Phenotype: A Systematic Review and Meta-Analysis, Journal of the American Medical Directors Association. (2019) 20, no. 12, 1647–1649.e2, 10.1016/j.jamda.2019.05.018. 31301987

[3] Schoufour J. D. , Erler N. S. , Jaspers L. et al., Design of a Frailty Index Among Community Living Middle-Aged and Older People: The Rotterdam Study, Maturitas. (2017) 97, 14–20, 10.1016/j.maturitas.2016.12.002. 28159055

[4] Struijk E. A. , Fung T. T. , Rodríguez-Artalejo F. et al., Protein Intake and Risk of Frailty Among Older Women in the Nurses’ Health Study, Journal of Cachexia, Sarcopenia and Muscle. (2022) 13, no. 3, 1752–1761, 10.1002/jcsm.12972. 35318829 PMC9178161

[5] Khanna A. K. , Motamedi V. , Bouldin B. et al., Automated Electronic Frailty Index-Identified Frailty Status and Associated Postsurgical Adverse Events, JAMA Network Open. (2023) 6, no. 11, 10.1001/jamanetworkopen.2023.41915. PMC10628731 37930697

[6] Jackson J. L. and Ganshert C. , Identifying Frailty as a Factor Associated With Adverse Outcomes in Cardiovascular Surgery-An Imperfect but Promising Science, JAMA Network Open. (2022) 5, no. 9, 10.1001/jamanetworkopen.2022.30970. 36083587

[7] Cheng M. H. and Chang S. F. , Frailty as a Risk Factor for Falls Among Community Dwelling People: Evidence From a Meta-Analysis, Journal of Nursing Scholarship. (2017) 49, no. 5, 529–536, 10.1111/jnu.12322. 28755453

[8] Vetrano D. , Zucchelli A. , Onder G. et al., Construction and Validation of a Frailty Index in Primary Care in Italy: The Health-Search Frailty Index, Innovation in Aging. (2021) 5, no. Supplement_1, 10.1093/geroni/igab046.2047.

[9] Feenstra M. , Oud , Jansen C. J. , Smidt N. , van Munster B. C. , and de Rooij S. E. , Reproducibility and Responsiveness of the Frailty Index and Frailty Phenotype in Older Hospitalized Patients, BMC Geriatrics. (2021) 21, no. 1, 10.1186/s12877-021-02444-y. PMC8447764 34535074

[10] He T. , Yan Y. , Wang D. , Peng T. , and Jin L. , Association of Dietary Health Indices With Frailty, BMC Public Health. (2025) 25, no. 1, 10.1186/s12889-025-22245-x. PMC11924830 40108600

[11] Chen C. , Winterstein A. G. , Fillingim R. B. et al., Body Weight, Frailty, and Chronic Pain in Older Adults: A Cross-Sectional Study, BMC Geriatrics. (2019) 19, no. 1, 10.1186/s12877-019-1149-4. PMC6534872 31126233

[12] Dai Z. , Wu Y. , Chen J. , Huang S. , and Zheng H. , Assessment of Relationships Between Frailty and Chronic Pain: A Bidirectional Two-Sample Mendelian Randomisation Study, Age and Ageing. (2024) 53, no. 1, 10.1093/ageing/afad256. 38251738

[13] Voshaar R. C. O. , Dimitriadis M. , vandenBrink R. H. S. et al., A 6-Year Prospective Clinical Cohort Study on the Bidirectional Association Between Frailty and Depressive Disorder, International Journal of Geriatric Psychiatry. (2021) 36, no. 11, 1699–1707, 10.1002/gps.5588. 34130356 PMC8596411

[14] Mannion H. and O’Caoimh R. , 261 Sleep and Frailty: Examining the Effects of Frailty on Sleep Disturbance in Hospitalised Older Adults, Age and Ageing. (2019) 48, no. Supplement_3, iii1–iii16, 10.1093/ageing/afz102.60.

[15] Jensen M. K. N. , O’Connell M. E. , and Mickleborough M. J. S. , Perceptions of Health, Cognition, and Pain Among Middle‐Aged and Older Adults With Migraine: A Population‐Based Cross‐Sectional Study Examining Findings From the Canadian Longitudinal Study on Aging, Headache. (2025) . 10.1111/head.14953 PMC12497939 40391574

[16] Togha M. , Karimitafti M. J. , Ghorbani Z. et al., Characteristics and sComorbidities of Headache in Patients over 50 Years of Age: A Cross-Sectional Study, BMC Geriatrics. (2022) 22, no. 1, 10.1186/s12877-022-03027-1. PMC8994908 35399063

[17] Berry J. K. M. and Drummond P. D. , Psychological Generators of Stress-Headaches, Journal of Behavioral Medicine. (2017) 41, no. 1, 109–121, 10.1007/s10865-017-9872-9. 28710564

[18] Bazargan M. , Comini J. , Kibe L. W. , Assari S. , and Cobb S. , Association Between Migraine and Quality of Life, Mental Health, Sleeping Disorders, and Health Care Utilization Among Older African American Adults, Journal of Racial and Ethnic Health Disparities. (2023) 11, no. 3, 1530–1540, 10.1007/s40615-023-01629-y. 37227684 PMC11101580

[19] Zhao Y. , Yi Y. , Zhou H. , Pang Q. , and Wang J. , The Burden of Migraine and Tension-Type Headache in Asia From 1990 to 2021, Journal of Headache and Pain. (2025) 26, no. 1, 10.1186/s10194-025-01990-9. PMC11892304 40065229

[20] Stovner J. L. , Hagen , Linde M. , and Steiner T. J. , The Global Prevalence of Headache: An Update, With Analysis of the Influences of Methodological Factors on Prevalence Estimates, Journal of Headache and Pain. (2022) 23, no. 1, 10.1186/s10194-022-01402-2. PMC9004186 35410119

[21] Olesen J. , Classification of Migraine and Tension-Type Headache, Cephalalgia. (2023) 43, no. 4, 10.1177/03331024221139238. 36935589

[22] Hernandez J. , Molina E. , Rodriguez A. et al., Headache Disorders: Differentiating Primary and Secondary Etiologies, Journal of Integrative Neuroscience. (2024) 23, no. 2, 10.31083/j.jin2302043. 38419454

[23] Kristoffersen E. S. , Lundqvist C. , and Russell M. B. , Illness Perception in People With Primary and Secondary Chronic Headache in the General Population, Journal of Psychosomatic Research. (2019) 116, 83–92, 10.1016/j.jpsychores.2018.12.001. 30654999

[24] Rozen T. D. , A New Subtype of Chronic Daily Headache Presenting in Older Women, Journal of Women’s Health. (2018) 27, no. 2, 203–208, 10.1089/jwh.2017.6460. 28945159

[25] Bottiroli S. , Greco R. , Franco V. et al., Peripheral Endocannabinoid Components and Lipid Plasma Levels in Patients With Resistant Migraine and Co-Morbid Personality and Psychological Disorders: A Cross-Sectional Study, International Journal of Molecular Sciences. (2024) 25, no. 3, 10.3390/ijms25031893. PMC10855606 38339171

[26] Tension-Type Headache, Nature Reviews Disease Primers. (2021) 7, no. 1. 10.1038/s41572-021-00263-4 33767173

[27] Cho S. J. , Song T. J. , and Chu M. K. , Sleep and Tension-Type Headache, Current Neurology and Neuroscience Reports. (2019) 19, no. 7, 10.1007/s11910-019-0953-8. 31144052

[28] Do T. P. , Heldarskard G. F. , Kolding L. T. , Hvedstrup J. , and Schytz H. W. , Myofascial Trigger Points in Migraine and Tension-Type Headache, Journal of Headache and Pain. (2018) 19, no. 1, 10.1186/s10194-018-0913-8. PMC6134706 30203398

[29] Kim B. S. , Chung P. W. , Kim B. K. et al., The Impact of Remission and Coexisting Migraine on Anxiety and Depression in Cluster Headache, Journal of Headache and Pain. (2020) 21, no. 1, 10.1186/s10194-020-01120-7. PMC7257141 32471362

[30] Dhaem O. B. de and Robbins M. S. , Cognitive Impairment in Primary and Secondary Headache Disorders, Current Pain and Headache Reports. (2022) 26, no. 5, 391–404, 10.1007/s11916-022-01039-5. 35239156 PMC8891733

[31] Almeida G. G. , Alkan S. , Hoepner R. , Euler A. , Diem L. , and Wagner F. , Chronic Fatigue and Headache in Post-COVID-19 Syndrome: A Radiological and Clinical Evaluation, Frontiers in Neurology. (2025) 15, 10.3389/fneur.2024.1526130. PMC11799671 39917434

[32] Yoo A. , Vgontzas A. , Chung J. et al., The Association Between Multidimensional Sleep Health and Migraine Burden Among Patients With Episodic Migraine, Journal of Clinical Sleep Medicine. (2023) 19, no. 2, 309–317, 10.5664/jcsm.10320. 36263856 PMC9892733

[33] Dodson C. A. and Goodloe J. M. , Headache and Weakness in a Young Adult Female, Journal of the American College of Emergency Physicians Open. (2022) 3, no. 4, 10.1002/emp2.12772. PMC9244344 35782347

[34] Xu X. , Ding N. , He J. et al., Associations Between Reversible and Potentially Reversible Cognitive Frailty and Falls in Community-Dwelling Older Adults in China: A Longitudinal Study, BMC Geriatrics. (2025) 25, no. 1, 10.1186/s12877-025-05872-2. PMC11971774 40188026

[35] Wu J. , Yuan X. , Zhao J. et al., Association of the Insulin Resistance Marker Triglyceride Glucose Index With Migraine: Results of a Cross-Sectional and Prospective Cohort Study, Journal of Oral & Facial Pain and Headache. (2025) 39, no. 1, 165–175, 10.22514/jofph.2025.017. PMC11934749 40129435

[36] Wang M. H. , Pan L. J. , Zhang Y. H. , Zhu H. Q. , Zhu X. B. , and Wang X. Q. , Prevalence and Risk Factors of Headache in Chinese With Stroke: A Cross-Sectional Study Based on CHARLS, Journal of Headache and Pain. (2024) 25, no. 1, 10.1186/s10194-024-01930-z. PMC11654334 39695395

[37] Qing L. , Zhu Y. , Feng L. et al., Exploring the Association Between Frailty Index and Low Back Pain in Middle-Aged and Older Chinese Adults: A Cross-Sectional Analysis of Data From the China Health and Retirement Longitudinal Study (CHARLS), BMJ Open. (2024) 14, no. 5, 10.1136/bmjopen-2024-085645. PMC11131124 38802272

[38] Kojima G. , Jivraj S. , Iliffe S. , Falcaro M. , Liljas A. , and Walters K. , Alcohol Consumption and Risk of Incident Frailty: The English Longitudinal Study of Aging, Journal of the American Medical Directors Association. (2019) 20, no. 6, 725–729, 10.1016/j.jamda.2018.10.011. 30503591

[39] Soltani S. , Jayedi A. , Ghoreishy S. et al., Alcohol Consumption and Frailty Risk: A Dose–Response Meta-Analysis of Cohort Studies, Age and Ageing. (2024) 53, no. 9, 10.1093/ageing/afae199. 39300899

[40] Jakubczyk A. , Wiśniewski P. , Trucco E. M. et al., The Synergistic Effect Between Interoceptive Accuracy and Alcohol Use Disorder Status on Pain Sensitivity, Addictive Behaviors. (2021) 112, 10.1016/j.addbeh.2020.106607. PMC7572764 32827968

[41] Davis-Martin R. E. , Polk A. N. , and Smitherman T. A. , Alcohol Use as a Comorbidity and Precipitant of Primary Headache: Review and Meta-Analysis, Current Pain and Headache Reports. (2017) 21, no. 10, 10.1007/s11916-017-0642-8. 28844083

[42] Imai N. and Kitamura E. , Differences in Clinical Features of Cluster Headache Between Drinkers and Nondrinkers in Japan, PLoS One. (2019) 14, no. 11, 10.1371/journal.pone.0224407. PMC6867697 31747412

[43] Arca K. N. and Singh R. B. H. , The Hypertensive Headache: A Review, Current Pain and Headache Reports. (2019) 23, no. 5, 10.1007/s11916-019-0767-z. 30874912

[44] Xiao G. , Huang Z. , Lan Q. et al., Evidence Supporting the Role of Hypertension in the Onset of Migraine, Journal of Translational Medicine. (2025) 23, no. 1, 10.1186/s12967-025-06187-x. PMC12023583 40275290

[45] Arosio B. , Ferri E. , Mari D. , Tobaldini E. , Vitale G. , and Montano N. , The Influence of Inflammation and Frailty in the Aging Continuum, Mechanism of Ageing and Development. (2023) 215, 10.1016/j.mad.2023.111872. 37689318

[46] Baş A. O. , Güner M. , Ceylan S. et al., Pan-Immune Inflammation Value; a Novel Biomarker Reflecting Inflammation Associated With Frailty, Aging Clinical and Experimental Research. (2023) 35, no. 8, 1641–1649, 10.1007/s40520-023-02457-0. 37289361

[47] Walker K. A. , Walston J. , Gottesman R. F. , Kucharska-Newton A. , Palta P. , and Windham B. G. , Midlife Systemic Inflammation is Associated With Frailty in Later Life: The ARIC Study, Journals of Gerontology. Series A, Biological Sciences and Medical Sciences. (2018) 74, no. 3, 343–349, 10.1093/gerona/gly045. PMC6376088 29534173

[48] Lauritano D. , Mastrangelo F. , D’Ovidio C. et al., Activation of Mast Cells by Neuropeptides: The Role of Pro-Inflammatory and Anti-Inflammatory Cytokines, International Journal of Molecular Sciences. (2023) 24, no. 5, 10.3390/ijms24054811. PMC10002992 36902240

[49] Wang Y. W. , Yang X. H. , Zheng X. H. et al., Unraveling the Relationship Between Inflammation and Cluster Headache, Frontiers in Neurology. (2025) 16, 10.3389/fneur.2025.1548522. PMC12003110 40248013

[50] Spekker E. , Tanaka M. , Szabó Á. , and Vécsei L. , Neurogenic Inflammation: The Participant in Migraine and Recent Advancements in Translational Research, Biomedicines. (2021) 10, no. 1, 10.3390/biomedicines10010076. PMC8773152 35052756

[51] Hu B. , Yu Y. , Dai Y. J. et al., Multi-Modal MRI Reveals the Neurovascular Coupling Dysfunction in Chronic Migraine, Neuroscience. (2019) 419, 72–82, 10.1016/j.neuroscience.2019.09.022. 31682827

[52] Han K. , Min J. , Lee M. et al., Neurovascular Coupling Under Chronic Stress is Modified by Altered Gabaergic Interneuron Activity, Journal of Neuroscience. (2019) 39, no. 50, 10081–10095, 10.1523/jneurosci.1357-19.2019. 31672788 PMC6978951

[53] Amarasekera A. T. , Chang D. , Schwarz P. , and Tan T. C. , Does Vascular Endothelial Dysfunction Play a Role in Physical Frailty and Sarcopenia? A Systematic Review, Age and Ageing. (2020) 50, no. 3, 725–732, 10.1093/ageing/afaa237. 33951149

[54] Tang Y. , Wang X. , Zhang S. et al., Pre-Existing Weakness is Critical for the Occurrence of Postoperative Cognitive Dysfunction in Mice of the Same Age, PLoS One. (2017) 12, no. 8, 10.1371/journal.pone.0182471. PMC5546624 28787017

[55] Krainik A. , Hund-Georgiadis M. , Zysset S. , and von Cramon D. Y. , Regional Impairment of Cerebrovascular Reactivity and BOLD Signal in Adults After Stroke, Stroke. (2005) 36, no. 6, 1146–1152, 10.1161/01.str.0000166178.40973.a7. 15879326

[56] de la Torre J. C. , Cerebral Hemodynamics and Vascular Risk Factors: Setting the Stage for Alzheimer’s Disease, Journal of Alzheimer’s Disease: JAD. (2012) 32, no. 3, 553–567, 10.3233/jad-2012-120793. 22842871

[57] Jin Y. , Zhou J. , Xu F. et al., Electroacupuncture Alleviates the Transition From Acute to Chronic Pain Through the p38 MAPK/TNF-α Signalling Pathway in the Spinal Dorsal Horn, Acupuncture in Medicine. (2021) 39, no. 6, 708–715, 10.1177/09645284211020766. 34308662

[58] Xu R. , Wang J. , Nie H. et al., Genome-Wide Expression Profiling by RNA-Sequencing in Spinal Cord Dorsal Horn of a Rat Chronic Postsurgical Pain Model to Explore Potential Mechanisms Involved in Chronic Pain, Journal of Pain Research. (2022) 15, 985–1001, 10.2147/jpr.s358942. 35411184 PMC8994637

[59] Inoue K. and Tsuda M. , Microglia in Neuropathic Pain: Cellular and Molecular Mechanisms and Therapeutic Potential, Nature Reviews Neuroscience. (2018) 19, no. 3, 138–152, 10.1038/nrn.2018.2. 29416128

