# Supplementary material for: Association Between the Frailty Index and Self‐Reported Headache in Middle‐Aged and Older Chinese Adults: An Analysis of the China Health and Retirement Longitudinal Study
Source: Pain Res Manag. 2026 Jul 14;2026:6632755. doi: 10.1155/prm/6632755 (PMC13369298; doi:10.1155/prm/6632755)
Supplement: Supplementary file 1 — Supporting Information Table S1. Baseline characteristics of participants included in and excluded from the missing‐data comparison sample. Table S2. List of health deficit items included in the frailty index. Table S3. Sensitivity analysis of the association between FI and headache after excluding participants with FI values above the 95th percentile. Table S4. STROBE Statement—checklist of items that should be included in reports of observational studies. [file PRM-2026-6632755-s001.docx]

**Table S1.** Baseline characteristics of participants included in and excluded from the missing-data comparison sample.

| **Characteristics** | **Total**  **(n=57416)** | **Include**  **(n=43907)** | **Exclude**  **(n=13509)** | ***P* value** | **SMD** |
| --- | --- | --- | --- | --- | --- |
| FI | 0.12 ± 0.11 | 0.11 ± 0.10 | 0.13 ± 0.13 | <.001 | 0.172 |
| Age (years) | 59.44 ± 10.39 | 60.10 ± 9.70 | 57.27 ± 12.17 | <.001 | 0.257 |
| BMI | 23.74 ± 3.82 | 23.72 ± 3.83 | 23.89 ± 3.76 | <.001 | 0.045 |
| Headache (%) |  |  |  | <.001 |  |
| No | 36473(84.77) | 30227(84.46) | 6246(86.33) |  |  |
| yes | 6551(15.23) | 5562(15.54) | 989(13.67) |  | 0.053 |
| Sex (%) |  |  |  | <.001 |  |
| Female | 29998(52.30) | 23224(52.92) | 6774(50.27) |  | 0.053 |
| Male | 27357(47.70) | 20657(47.08) | 6700(49.73) |  |  |
| Marital status (%) |  |  |  | 0.58 |  |
| Married | 49845(86.96) | 38178(87.00) | 11667(86.81) |  |  |
| Non Married | 7475(13.04) | 5703(13.00) | 1772(13.19) |  | 0.006 |
| Education (%) |  |  |  | <.001 |  |
| High school or above | 7049(12.81) | 5175(11.79) | 1874(16.83) |  |  |
| Illiterate | 14493(26.34) | 11644(26.53) | 2849(25.58) |  | 0.022 |
| Junior high school | 33477(60.85) | 27063(61.67) | 6414(57.59) |  | 0.083 |
| Residence place (%) |  |  |  | <.001 |  |
| Rural | 34169(59.52) | 27244(62.06) | 6925(51.28) |  | 0.217 |
| Urban | 23236(40.48) | 16657(37.94) | 6579(48.72) |  |  |
| Smoke (%) |  |  |  | 0.35 |  |
| No | 39725(74.38) | 30486(74.48) | 9239(74.05) |  |  |
| Yes | 13685(25.62) | 10448(25.52) | 3237(25.95) |  | 0.010 |
| Drink (%) |  |  |  | <.001 |  |
| No | 37336(65.70) | 28922(66.14) | 8414(64.23) |  |  |
| Yes | 19492(34.30) | 14806(33.86) | 4686(35.77) |  | 0.040 |
| Hypertension (%) |  |  |  | <.001 |  |
| No | 33673(60.72) | 25772(58.98) | 7901(67.23) |  |  |
| Yes | 21779(39.28) | 17927(41.02) | 3852(32.77) |  | 0.171 |
| Diabetes mellitus (%) |  |  |  | <.001 |  |
| No | 47927(88.11) | 38074(87.62) | 9853(90.07) |  |  |
| Yes | 6467(11.89) | 5381(12.38) | 1086( 9.93) |  | 0.078 |
| Dyslipidemia (%) |  |  |  | <.001 |  |
| No | 40506(75.05) | 32159(74.32) | 8347(77.99) |  |  |
| Yes | 13465(24.95) | 11110(25.68) | 2355(22.01) |  | 0.086 |

Mean ± SD for continuous variables: the P value was analyzed via ANOVA. (%) for categorical variables: the P value was analyzed via the chi-square test.

**Table S2.** List of health deficits items included in the frailty index.

| **Type of deficit** | **Item** | **Variables** | **Cut-off point** |
| --- | --- | --- | --- |
| Activities of daily living | 1 | Bathing | No=0; a little=0.33; need help=0.67; Yes=1 |
|  | 2 | Dressing | No=0; a little=0.33; need help=0.67; Yes=1 |
|  | 3 | Use of toilet | No=0; a little=0.33; need help=0.67; Yes=1 |
|  | 4 | Transferring | No=0; a little=0.33; need help=0.67; Yes=1 |
|  | 5 | Continence | No=0; a little=0.33; need help=0.67; Yes=1 |
|  | 6 | Eating | No=0; a little=0.33; need help=0.67; Yes=1 |
| Instrumental Activities of dailyliving | 7 | Cooking | No=0; a little=0.33; need help=0.67; Yes=1 |
|  | 8 | Shopping | No=0; a little=0.33; need help=0.67; Yes=1 |
|  | 9 | Doing household | No=0; a little=0.33; need help=0.67; Yes=1 |
|  | 10 | Taking medicine | No=0; a little=0.33; need help=0.67; Yes=1 |
|  | 11 | Managing money | No=0; a little=0.33; need help=0.67; Yes=1 |
| Physical functional limitations | 12 | Lift a weight of 5kg | No=0; a little=0.33; need help=0.67; Yes=1 |
|  | 13 | Walking 1 km | No=0; a little=0.33; need help=0.67; Yes=1 |
|  | 14 | Walking 100 m | No=0; a little=0.33; need help=0.67; Yes=1 |
|  | 15 | Stooping,kneeling, orcrouching | No=0; a little=0.33; need help=0.67; Yes=1 |
|  | 16 | Able to stand up from sitting | No=0; a little=0.33; need help=0.67; Yes=1 |
|  | 17 | Able to pick up a coin from atable | No=0; a little=0.33; need help=0.67; Yes=1 |
|  | 18 | Running or jogging about 1km | No=0; a little=0.33; need help=0.67; Yes=1 |
|  | 19 | Reaching or extend arms | No=0; a little=0.33; need help=0.67; Yes=1 |
|  | 20 | Climbing several flights of stairs without resting | No=0; a little=0.33; need help=0.67; Yes=1 |
| Chronic disease | 21 | Chronic lung diseases (Chronic bronchitis, emphysema) | Yes=1; no=0 |
|  | 22 | Asthma | Yes=1; no=0 |
|  | 23 | stroke | Yes=1; no=0 |
|  | 24 | CVD | Yes=1; no=0 |
|  | 25 | Gastric or duodenal ulcer | Yes=1; no=0 |
|  | 26 | Kidney disease | Yes=1; no=0 |
|  | 27 | Liver disease | Yes=1; no=0 |
|  | 28 | Memory related disease (Dementia, brain atrophy, and Parkinson's disease) | Yes=1; no=0 |
|  | 29 | Emotional, nervous, or psychiatric problems | Yes=1; no=0 |
| Mental health | 30 | Feel depressed | Always=1; often=0.67; sometimes=0.33; Seldom or never=0 |
|  | 31 | Feel fearful | Always=1; often=0.67; sometimes=0.33; Seldom or never=0 |
|  | 32 | Feel happy | Always=0; often=0.33; sometimes=0.67; Seldom or never=1 |
|  | 33 | Feel everything was an effort | Always=1; often=0.67; sometimes=0.33; Seldom or never=0 |
|  | 34 | Feel could not get "going" | Always=1; often=0.67; sometimes=0.33; Seldom or never=0 |
| Subjective functioning | 35 | Self-rated health | Very good=0; good=0.25; average=0.5; bad=0.75; very bad=1 |

**Table S3.** Sensitivity analysis of the association between FI and headache after excluding participants with FI values above the 95th percentile.

| **Characteristic** | **Model 1** | | **Model 2** | | **Model 3** | |
| --- | --- | --- | --- | --- | --- | --- |
|  | ***OR* (95% CI)** | ***P*-value** | ***OR* (95% CI)** | ***P*-value** | ***OR* (95% CI)** | ***P*-value** |
| FI | 1.176(1.170,1.182) | <0.001 | 1.185(1.178,1.192) | <0.001 | 1.185(1.178,1.192) | <0.001 |
| FI groups |  | | | | | |
| Q1 | reference |  | reference |  | reference |  |
| Q2 | 2.834(2.342, 3.429) | <0.001 | 2.748(2.270, 3.328) | <0.001 | 2.745(2.267,3.324) | <0.001 |
| Q3 | 7.262(6.131, 8.601) | <0.001 | 7.035(5.931, 8.345) | <0.001 | 6.988(5.891,8.290) | <0.001 |
| Q4 | 26.654(22.668,31.341) | <0.001 | 27.248(23.087,32.159) | <0.001 | 27.056(22.915,31.946) | <0.001 |
| *P* for trend |  | <0.001 |  | <0.001 |  | <0.001 |

Note: For continuous FI, ORs are presented per 0.1-point increase.

Model 1: No covariates were adjusted.

Model 2: Age, sex, residence, marital status, educational attainment, BMI were adjusted.

Model 3: Age, sex, residence, marital status, educational attainment, BMI, smoking status, drinking status, hypertension, diabetes mellitus, dyslipidemia were adjusted.

**Table S4.** STROBE Statement—checklist of items that should be included in reports of observational studies.

|  | Item No. | Recommendation | Page  No. |
| --- | --- | --- | --- |
| **Title and abstract** | 1 | (*a*) Indicate the study’s design with a commonly used term in the title or the abstract | Page 1-2 |
|  |  | (*b*) Provide in the abstract an informative and balanced summary of what was done and what was found | Page 2 |
| Introduction | | | |
| Background/rationale | 2 | Explain the scientific background and rationale for the investigation being reported | Page 3-4 |
| Objectives | 3 | State specific objectives, including any prespecified hypotheses | Page 4 |
| Methods | | | |
| Study design | 4 | Present key elements of study design early in the paper | Page 4-5 |
| Setting | 5 | Describe the setting, locations, and relevant dates, including periods of recruitment, exposure, follow-up, and data collection | Page 4-5 |
| Participants | 6 | (*a*) Give the eligibility criteria, and the sources and methods of selection of participants | Page 5-6 |
| Variables | 7 | Clearly define all outcomes, exposures, predictors, potential confounders, and effect modifiers. Give diagnostic criteria, if applicable | Page 6-7 |
| Data sources/ measurement | 8* | For each variable of interest, give sources of data and details of methods of assessment (measurement). Describe comparability of assessment methods if there is more than one group | Page 8-9 |
| Bias | 9 | Describe any efforts to address potential sources of bias | Not applicable |
| Study size | 10 | Explain how the study size was arrived at | Not applicable |

Continued on next page

| Quantitative variables | 11 | Explain how quantitative variables were handled in the analyses. If applicable, describe which groupings were chosen and why |  | Page 8 |
| --- | --- | --- | --- | --- |
| Statistical methods | 12 | (*a*) Describe all statistical methods, including those used to control for confounding |  | Page 8-9 |
|  |  | (*b*) Describe any methods used to examine subgroups and interactions |  | Page 8-9 |
|  |  | (*c*) Explain how missing data were addressed |  | Page 5 |
|  |  | (*d*) If applicable, describe analytical methods taking account of sampling strategy |  | Page 8-9 |
|  |  | (*e*) Describe any sensitivity analyses |  | Page 8-9 |
| Results | | | | |
| Participants | 13* | (a) Report numbers of individuals at each stage of study—eg numbers potentially eligible, examined for eligibility, confirmed eligible, included in the study, completing follow-up, and analysed |  | Page 5-6 |
|  |  | (b) Give reasons for non-participation at each stage |  | Page 5-6 |
|  |  | (c) Consider use of a flow diagram |  | Page 6 |
| Descriptive data | 14* | (a) Give characteristics of study participants (eg demographic, clinical, social) and information on exposures and potential confounders |  | Page 6-7 |
|  |  | (b) Indicate number of participants with missing data for each variable of interest |  | Page 5 |
| Outcome data | 15* | Report numbers of outcome events or summary measures |  | Page 5 |
| Main results | 16 | (*a*) Give unadjusted estimates and, if applicable, confounder-adjusted estimates and their precision (eg, 95% confidence interval). Make clear which confounders were adjusted for and why they were included |  | Page 11 |
|  |  | (*b*) Report category boundaries when continuous variables were categorized |  | Page 11 |
|  |  | (*c*) If relevant, consider translating estimates of relative risk into absolute risk for a meaningful time period |  | Not applicable |

Continued on next page

| Other analyses | 17 | Report other analyses done—eg analyses of subgroups and interactions, and sensitivity analyses |  | Page 12-14 |
| --- | --- | --- | --- | --- |
| Discussion | | | | |
| Key results | 18 | Summarise key results with reference to study objectives |  | Page 14 |
| Limitations | 19 | Discuss limitations of the study, taking into account sources of potential bias or imprecision. Discuss both direction and magnitude of any potential bias |  | Page 16-17 |
| Interpretation | 20 | Give a cautious overall interpretation of results considering objectives, limitations, multiplicity of analyses, results from similar studies, and other relevant evidence |  | Page 17 |
| Generalisability | 21 | Discuss the generalisability (external validity) of the study results |  | Page 16 |
| Other information | |  | | |
| Funding | 22 | Give the source of funding and the role of the funders for the present study and, if applicable, for the original study on which the present article is based |  | Not applicable |

*Give information separately for cases and controls in case-control studies and, if applicable, for exposed and unexposed groups in cohort and cross-sectional studies.

**Note:** An Explanation and Elaboration article discusses each checklist item and gives methodological background and published examples of transparent reporting. The STROBE checklist is best used in conjunction with this article (freely available on the Web sites of PLoS Medicine at http://www.plosmedicine.org/, Annals of Internal Medicine at http://www.annals.org/, and Epidemiology at http://www.epidem.com/). Information on the STROBE Initiative is available at www.strobe-statement.org.
